# A data-driven approach to implementing the HPTN 094 complex intervention INTEGRA in local communities

**DOI:** 10.1186/s13012-024-01363-x

**Published:** 2024-06-03

**Authors:** Laramie R. Smith, Amaya Perez-Brumer, Melanie Nicholls, Jayla Harris, Qiana Allen, Alan Padilla, Autumn Yates, Eliza Samore, Rebecca Kennedy, Irene Kuo, Jordan E. Lake, Cecile Denis, David Goodman-Meza, Peter Davidson, Steve Shoptaw, Nabila El-Bassel, for the HPTN 094 study protocol team

**Affiliations:** 1 School of Medicine, University of California San Diego, 9500 Gilman Drive #0507, La Jolla, CA 92093-0507 USA; 2 Dalla Lana School of Public Health, University of Toronto, Toronto, Canada; 3 School of Social Work, San Diego State University, San Diego, USA; 4 HIV Prevention Trials Network, Family Health International 360, Durham, USA; 5 UTHealth Houston McGovern School of Medicine, Houston, USA; 6 Columbia University, ICAP, Mailman School of Public Health, New York, USA; 7 George Washington University Milken Institute School of Public Health, Washington, DC USA; 8 Center for Behavioral and Addiction Medicine at UCLA, Los Angeles, USA; 9 University of Pennsylvania, Perelman School of Medicine, Philadelphia, USA; 10 Division of Infectious Diseases at UCLA, Los Angeles, USA; 11 Department of Family Medicine, David Geffen School of Medicine at UCLA, Los Angeles, USA; 12 School of Social Work, Columbia University, New York, USA

**Keywords:** Implementation science, People who inject drugs, HIV prevention, Complex interventions, Mobile health, Health equity, Integrated care, Practical Robust Implementation Sustainability Model (PRISM)

## Abstract

**Background:**

HIV burden in the US among people who inject drugs (PWID) is driven by overlapping syndemic factors such as co-occurring health needs and environmental factors that synergize to produce worse health outcomes among PWID. This includes stigma, poverty, and limited healthcare access (e.g. medication to treat/prevent HIV and for opioid use disorder [MOUD]). Health services to address these complex needs, when they exist, are rarely located in proximity to each other or to the PWID who need them. Given the shifting drug use landscapes and geographic heterogeneity in the US, we evaluate a data-driven approach to guide the delivery of such services to PWID in local communities.

**Methods:**

We used a hybrid, type I, embedded, mixed method, data-driven approach to identify and characterize viable implementation neighborhoods for the HPTN 094 complex intervention, delivering integrated MOUD and HIV treatment/prevention through a mobile unit to PWID across five US cities. Applying the PRISM framework, we triangulated geographic and observational pre-implementation phase data (epidemiological overdose and HIV surveillance data) with two years of implementation phase data (weekly ecological assessments, study protocol meetings) to characterize environmental factors that affected the viability of implementation neighborhoods over time and across diverse settings.

**Results:**

Neighborhood-level drug use and geographic diversity alongside shifting socio-political factors (policing, surveillance, gentrification) differentially affected the utility of epidemiological data in identifying viable implementation neighborhoods across sites. In sites where PWID are more geographically dispersed, proximity to structural factors such as public transportation and spaces where PWID reside played a role in determining suitable implementation sites. The utility of leveraging additional data from local overdose and housing response systems to identify viable implementation neighborhoods was mixed.

**Conclusions:**

Our findings suggest that data-driven approaches provide a contextually relevant pragmatic strategy to guide the real-time implementation of integrated care models to better meet the needs of PWID and help inform the scale-up of such complex interventions. This work highlights the utility of implementation science methods that attend to the impact of local community environmental factors on the implementation of complex interventions to PWID across diverse drug use, sociopolitical, and geographic landscapes in the US.

**Trial registration:**

ClincalTrials.gov, Registration Number: NCT04804072. Registered 18 February 2021.

Contributions to literature

- Research shows that healthcare delivered in mobile units can expand service reach to underserved communities.
- Few studies describe how or if a process was used to optimize the reach and adoption of such mobilized services.
- This analysis attends to factors in the external environment that expand beyond traditional surveillance data to inform timely adaptations and expand the reach of healthcare delivered via mobile units in underserved communities.
- Our findings contribute to the literature and suggest that data-driven approaches may provide a pragmatic strategy to guide the real-time implementation of integrated care models in local communities with heterogeneous external environments.

## Background

Approximately 3.7 million people who inject drugs (PWID) in the United States (US) represent 1.46% of the US population [1], and PWID account for 7% of new HIV acquisitions annually [2]. The domestic HIV burden among PWID is driven by interconnected syndemic factors, including limited access to healthcare and medication for opioid use disorder (MOUD), poverty, poly-substance use, and unmet mental health needs [3, 4]. The health impacts of these syndemic factors are further amplified by the criminalization of drug use [5, 6] and stigma towards PWID within systems of care, which contribute to the disruption or discontinuation of healthcare services, including limited access to HIV-related medications and MOUD [5, 7]; hindering treatment adherence and retention.

While the introduction of antiretroviral therapy (ART) has greatly improved the life expectancy of people with HIV, the life expectancy of PWID with HIV has remained stagnant [8]. Similarly, pre-exposure prophylaxis (PrEP) is recommended for PWID to prevent HIV acquisition [9], but PrEP prescription and uptake remain alarmingly low [10]. The changing US drug landscape, precipitated by the increased availability of prescription opioids and subsequent dominance of fentanyl across the US, has accelerated the potential for higher rates of HIV transmission and fatal overdose among PWID [11, 12]. MOUD is a highly effective HIV prevention and health promotion strategy among PWID, reducing risk of overdose and HIV-related harms [13, 14], but access and uptake of MOUD remain under-utilized across the US [15, 16].

PWID not engaged in MOUD, regardless of HIV status, face significant challenges in accessing comprehensive care [17–19]. Many traditional healthcare settings provide separate and fragmented care for opioid use (methadone, buprenorphine), HIV (ART, PrEP), and primary care services (STI testing and treatment, hepatitis testing and treatment, diagnosis and treatment of chronic conditions). Neighborhood-level factors related to available public transportation and the geographic location of fragmented services in proximity to where PWID reside may further impede service access. The lack of integration and accessibility of healthcare services for PWID with multiple health needs has contributed to HIV outbreaks in various parts of the US, such as Indiana, Massachusetts, Washington State, and West Virginia [20–22]. As such, international guidelines recommend integrating ART, PrEP, and MOUD evidence-based interventions for the treatment and prevention of HIV and substance use disorders [23].

Increasingly, implementation science (IS) is being used to aid in the planning and successful delivery of complex evidence-based interventions and their adoption among PWID in the US and abroad. Prior MOUD and harm reduction research has examined implementation facilitators and barriers within the organizational healthcare and social service systems [24–32], workforce capacity [33], financing, and policy contexts [34, 35]. In the context of HIV and substance use, IS frameworks and methods have been leveraged in the US to articulate organization and patient perspectives of harm reduction interventions for PWID with HIV [36], improve the success of HIV prevention interventions by accounting for the unique injection contexts on HIV risk behaviors among PWID [37], and help to expand harm reduction interventions via pharmacy services directed to PWID [38].

The goal of the HIV Prevention Trials Network (HPTN) 094 multisite trial is to determine the effectiveness of delivering a complex intervention, integrated MOUD and HIV care, to PWID through a mobile unit in their local communities to improve treatment initiation and retention [39]. Given the evolving US drug use landscape and the sociopolitical and geographical diversity, this assessment was designed to evaluate a data-driven IS approach to guide the delivery of such services to PWID across five US cities affected by intersecting HIV and opioid epidemics. Developing flexible, real-time, data-driven approaches that leverage multiple data sources to account for how contextual factors in the environment affect the viability of delivering integrated care to PWID in local communities is imperative to expanding the reach and impact of an evidence-based public health response.

## Methods

As a hybrid, type 1 effectiveness implementation study [40], we evaluate the effectiveness of a complex intervention, INTEGRA, which consists of integrated HIV and MOUD care delivered through a mobile unit paired with peer navigation to PWID. The primary clinical focus is to determine if PWID randomized to INTEGRA have better retention on MOUD and increased retention on HIV treatment (PrEP/ART) at week 26 compared to PWID randomized to the peer navigation active control arm at week 26 [39]. We do not report on clinical effectiveness outcomes as the trial is ongoing. This paper examines the embedded, mixed method implementation evaluation that included a data-driven approach to contextualize and guide the pre-implementation planning and real-time delivery of INTEGRA within local neighborhoods across five US cities, including New York City (NYC), NY; Philadelphia, PA; Washington DC (DC); Houston, TX; and Los Angles (LA), California from May 2020 to June 2025.

### Theoretical model

**Fig. 1:**
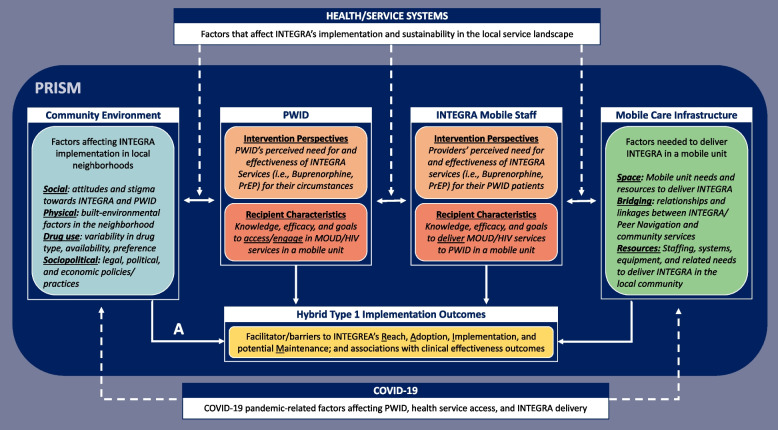
PRISM applied to the HPTN 094 implementation evaluation. Figure 1 depicts the conceptual linkages between the PRISM determinants on HPTN 094 implementation and clinical outcomes of interest

The implementation evaluation is guided by the Practical Robust Implementation Sustainability Model (PRISM; Fig. 1), [41] a multilevel framework that aims to move research findings towards healthcare practice [42]. PRISM’s four theoretical domains guided our implementation assessments of (i) the patient (i.e., PWID) and healthcare organizational (i.e., mobile unit) perceptions of INTEGRA’s integrated evidence-based interventions (i.e., buprenorphine, ART/PrEP), (ii) characteristics of PWID and the mobile unit staff that affect the delivery of integrated care, (iii) factors in the external community environment that can influence how integrated care was delivered by the mobile unit and accessed by PWID, and (iv) systems-level factors and infrastructure needs that affect or are affected by the delivery of integrated care through the mobile unit. We further assessed linkages between PRISM determinants, the local healthcare/public health systems, and the COVID-19 pandemic. We developed this process to prospectively examine the role these determinants had on implementation outcomes when conducting the RCT (e.g., appropriateness or viability of local implementation neighborhoods) and on subsequent observed clinical outcomes among PWID following the RCT evaluation (e.g., adoption and maintenance of ART/PrEP and MOUD treatment at week 26 post-baseline).

This analysis draws on multiple data sources to account for how contextual factors in the community environment (Qual) affect the viability of delivering INTEGRA in local neighborhoods (Quant) (Fig. 1, Path A). The viability of implementation neighborhoods is characterized as the proportion of participants enrolled within a site’s implementation neighborhood. High enrollment neighborhoods were classified as neighborhoods where ≥30% of participants from a site were enrolled, and characteristics of these neighborhoods were compared within and across study sites.

### Participants and procedures

Implementation data for this analysis were prospectively collected from HPTN 094 study protocol meetings and study site staff responsible for the day-to-day implementation of the integrated intervention to PWID within the selected implementation neighborhoods. This analysis leverages data collected in the pre-implementation phase (11-2020 to 04-2021) and the first two years of the implementation phase (05-2021 to 04-2023), spanning the initial years of the COVID-19 pandemic. All study procedures are approved by a central institutional review board (Advarra). As non-human subject observational data, written informed consent was not required for the data used in this analysis. The HPTN 094 protocol is registered at ClinicalTrials.gov (NCT04804072).

### Measures

In the pre-implementation phase, a site-level landscape analysis documented the baseline status of the local HIV and opioid epidemics via local HIV incidence and overdose surveillance data. Using Google Maps™, each site documented the boundaries of the neighborhoods identified in the surveillance data as co-affected by the HIV and opioid epidemics. Sites geotagged the mobile units home base and services within the vicinity of each neighborhood that reflected proximity to (a) community services for HIV, MOUD, harm reduction, and non-emergency primary care services, and (b) institutions that may discharge PWID back into the community (i.e., emergency rooms, jails, prisons) to characterize the existing service landscape in each of the priority neighborhoods. Each site used the maps to guide the selection of local neighborhoods with the greatest needs where participant recruitment and intervention delivery activities would occur.

Throughout the implementation phase, field staff from each site completed a weekly brief ecological assessment to document the dates and cross-streets where INTEGRA was implemented. Open-ended prompts were used to elicit neighborhood-level factors affecting their ability to reach eligible PWID (i.e., *What is affecting the recruitment and retention of PWID in the neighborhood?*) and provide integrated care in the mobile unit (i.e., *What is affecting how the intervention is delivered or accessed in the neighborhood?*) including reasons for refusing to enroll in the intervention (i.e., *Why did anyone decline to participate in the study?*) and obtain any supplemental observations the field site felt affected the conduct of the study (e.g., *Is there anything else the implementation science team should know?*). Neighborhood boundaries were added to Google Maps™ when a site identified a new implementation neighborhood not identified in the pre-implementation phase. The cross-streets at which the mobile unit delivered the intervention were geocoded in Google Maps™. Staffs’ qualitative ecological observations were chronologically aggregated by implementation neighborhood, then uploaded quarterly as qualitative data for coding and analysis. Quantitative participant enrollment data was linked to the respective sites’ geographic location by enrollment date.

### Analysis

Our mixed methods implementation evaluation was conducted concurrently, with triangulation occurring in the analysis to draw on the multiple data sources (Quan→ ←Qual) [43–45]. This analytic approach allowed for an inductive and deductive assessment of how contextual factors in the community environment affect the viability of delivering INTEGRA in local neighborhoods (Fig. 1, Path A).

Quantitative data sources characterize the viability of implementation neighborhoods in relation to the local HIV and overdose epidemics and the geolocation of where INTEGRA was delivered (i.e., mobile unit cross-streets, field days, participant enrollments). Descriptive statistics characterize data used to select priority neighborhoods in the pre-implementation phase. For sites where surveillance data reported the total new HIV diagnoses or overdose deaths, the rate per 100,000 population was calculated by dividing the total number of cases by the neighborhood population and multiplied by 100,000. The geotagged implementation cross-streets characterized the geographic coverage of the intervention and were used to calculate the average driving distance (one way) from the units’ home base to implementation sites within each neighborhood. For each neighborhood, we assessed the total number of field days at that implementation site, and its overall viability was computed as the proportion of participants enrolled within that neighborhood out of the total number of participants enrolled at that site.

Qualitative data sources contextualized social, physical, drug use, and sociopolitical characteristics that affected INTEGRA implementation in local neighborhoods. Qualitative field notes taken by the first author (LS) from the HPTN 094 protocol meetings and observations documented during the pre-implementation landscape analysis and via weekly ecological observations during the first two years of the implementation phase. These data were primarily coded manually in Excel by the first author (LS). To improve the validity and accuracy, emerging observations were iteratively discussed during monthly IS calls with other team members, including site investigators and staff, to solidify convergence, resolve discrepancies, and describe what factors affected the viability of an implementation neighborhood or informed a site’s decision to abandon or onboard new implementation neighborhoods.

Quantitative and qualitative data triangulation was a multi-step process that utilized the neighborhood maps for each site as our reference point. Analysis was guided by PRISM and the quantitative data was presented on maps via color-coding to identify high-enrollment neighborhoods and the degree to which new neighborhoods were added during the implementation phase. The maps provided a geographic gestalt for assessing the density and dispersion of implementation neighborhoods across sites and the degree to which the mobile unit needed to move within a given neighborhood. Next, qualitative data, including memos on observed social, physical, drug use, and sociopolitical environmental contexts, were color-coded as facilitators or barriers and pinned to printed copies of the maps. This was an inductive and deductive approach as PRISM guided it to facilitate the ability to compare and contrast observations within and across sites while also learning from the unique contextual factors affecting neighborhood viability. Finally, quotes reflective of our primary observations were added to illustrate the interplay of multiple contextual factors in our data-driven process.

## Results

**Fig. 2:**
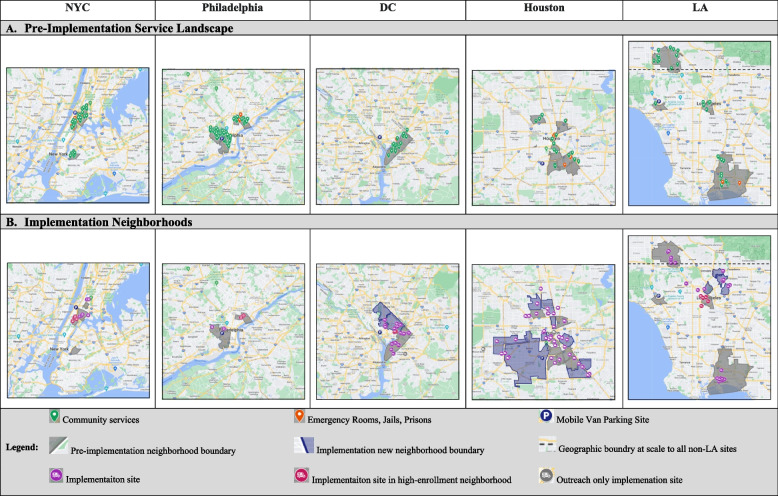
Geographic Coverage of the HPTN 094 Complex Intervention Data-Driven Implementation Mapping Process. Figure 2 depicts the data-driven process to identify priority implementation neighborhoods in the pre-implementation phase and characterize viable implementation neighborhoods in the two-year implementation phase

Findings are organized by pre-implementation neighborhood selection (Fig. 2A) and the range of geographic area covered across neighborhoods in the implementation phase (Fig. 2B). We report on the contextual barriers and facilitators that characterized the viability of implementation neighborhoods and the benefit of utilizing additional systems to guide real-time implementation decisions beyond the pre-implementation surveillance data.

### Pre-implementation neighborhood selection for delivering INTEGRA to PWID

Guided by HIV and overdose surveillance data, a total of 24 priority neighborhoods were identified across all sites, 10 of which were in Houston (Table 1). Across priority neighborhoods, HIV incidence was highest in Downtown LA (1,106 per 100,000), followed by Houston (Greater Third Ward=562 and Kashmere Gardens=508 per 100,000, respectively). Comparatively, the highest overdose death rates were observed in Philadelphia’s Kensington neighborhood (327.0 per 100,000), followed by Downtown LA (175.5 per 100,000) and the Sunnyside neighborhood in Houston (151.7 per 100,000).

**Table 1:** Pre-implementation priority neighborhood characteristics

Study Site	Neighborhood	Zip Code	Population Size ^a^	Overdose Deaths per 100,000	HIV Incidence per 100,000	No. of Community Services	No. of ERs, Jails, Prisons
NYC ^b^	Bushwick/Williamsburg	11206	156,505	23.8	37.8	12	1
	Crotona-Tremont	10457	172,000	43.8	54.6	6	2
	East Harlem	10035	98,500	35.5	37.6	13	3
	Melrose/Mott Haven	10445	164,000	40.1-55.0	31.9	22	5
Philadelphia ^c^	Kensington	19133, 19134	88,928	327	211	37	2
	South Philadelphia	19145, 19146	85,496	91	166	18	1
	West Philadelphia	19139, 19143	110,117	81	247	20	2
DC ^d^	Ward 7	20019	76,255	69.5	433	9	1
	North Ward 8	20020	78,513	94.3	485	7	0
	South Ward 8	20032	78,513	94.3	486	5	1
Houston ^e^	Central Northwest	77018	27,398	54.7	158	3	0
	First Ward	77002	20,787	67.3	421	2	3
	Greater OST	77021	28,921	107.2	457	1	0
	Greater Third Ward	77004	34,702	86.5	562	2	1
	Greenway Plaza	77046	24,673	121.6	----^**f**^	0	0
	Golfcrest/Belfort	77087	38,026	65.7	206	2	1
	Hobby Airport	77061	24,914	80.3	269	2	1
	Kashmere Gardens	77026	23,249	89.3	508	1	1
	South Park	77033	29,233	71.8	401	0	1
	Sunnyside	77051	17,139	151.7	457	2	0
LA ^g^	Downtown LA	90013	13,413	175.5	1106^**g**^	8	0
	Long Beach	90807	456,154	13.2	22	13	2
	The Valley	91405	127,440	24.3	125^**g**^	15	0
	West LA	90291	32,773	15.5	67^**g**^	1	0

^a^2020 US Census Data

^b^NYC reported 2019 HIV [42] and 2019-20 overdose surveillance data [43, 44]

^c^Philadelphia reported 2018 HIV surveillance data from AIDSVu accessed January 2021 [45] and overdose surveillance data July 2019-Aug 2020 [46, 47]

^d^DC reported 2020 HIV Surveillance data from AIDSVu by zip code [48] and overdose deaths recorded in 2020 by Ward [49]

^e^Houston reported 2018 HIV surveillance from AIDSVu accessed January 2021 [45] and overdose deaths recorded in 2020 [50]

^f^Data not reported to protect privacy of a small number of cases and/or small population

^g^LA reported 2018 HIV surveillance data from AIDSVu accessed January 2021 and 2020 surveillance data from LA County [51] and overdose deaths recorded in 2019 [52]

Priority neighborhoods differed regarding the number of community services providing HIV, MOUD, harm reduction, or primary care services within the neighborhood boundaries (Fig. 2B). Houston had substantially lower access to services within priority neighborhoods (Range: 0-3 community services per neighborhood; Table 1). Comparatively, priority neighborhoods in NYC, Philadelphia, and some areas of LA had greater access to services (Range 12-37), with the highest number of services documented in Philadelphia’s Kensington neighborhood. Probative observations identified that the higher number of services in Philadelphia was due to a standing order for all pharmacies to provide naloxone, substantially increasing access to harm reduction services across the city. Notably, systems where PWID may re-enter the community (i.e., emergency rooms, jails, prisons) were highest in NYC’s Melrose/Mott Haven and East Harlem Neighborhoods and Houston’s First Ward neighborhood.

### Implementation neighborhood geographic coverage and enrollment characteristics

Of the 24 priority neighborhoods identified in the pre-implementation landscape analysis, 18 were initiated in the implementation phase, as were 17 new neighborhoods not previously identified through the landscape analysis. Six priority neighborhoods were never initiated, including three in NYC and Philadelphia due to existing high-enrollment neighborhoods, and three in Houston with limited mobile unit parking options (i.e., First Ward) or because other viable neighborhoods emerged via local response systems described below.

As observed in Fig. 2B, the geographic density of implementation sites within neighborhoods is greatest in NYC and Philadelphia, where open drug markets provided concentrated areas to reach eligible PWID (Table 2). Both East Harlem and Kensington high enrollment neighborhoods are characterized as well-known areas to procure drugs, so the need to move the mobile unit within these neighborhoods was minimal. Notably, the implementation sites in Kensington covered an area less than 1 mile in length but accounted for 398 of the 410 days the mobile unit was in the field. Field staff determined after 12 days that PWID identified in West Philadelphia were also frequenting the Kensington neighborhood, where they expressed preference for engaging with the mobile unit. Similarly, NYC teams described the need to move the mobile van a few blocks to reach PWID who can’t cross specific streets due to drug turf boundaries:

*Identified a few places to move to keep things Fresh! Gated area called ‘Little Jungle’ an encampment where folks sleep at night and use two blocks from where the van* (mobile unit) *is parked. Some folks just don’t make it that far so bringing the truck over to this street… A block up from little Jungle is Big Jungle – runs under the L – another population that doesn’t make* (it) *two blocks down to our truck* (mobile unit) *so thinking to try this area out after next week.”* (Steering Committee Meeting 09/09/2022)

**Table 2:** Implementation Neighborhood PWID Enrollment Characteristics (*N*=358)^a^

Study Site	No. PWID Enrolled	Neighborhood	No. of Days per Neighborhood	Driving Distance Mean (SD)	% PWID Enrolled
NYC	*n*=80	Crotona- Tremont	--	--	--
		Bushwick/Williamsburg	--	--	--
		**East Harlem**	**177**	**3.86 mi (0.16)**	**58%**
		Melrose/Mott Haven	70	3.17 mi (0.69)	19%
	^b^	Fordham/Bronx Park	42	3.99 mi (0.12)	24%
Philadelphia	*n*=90	**Kensington**	**398**	**11.87 mi (0.05)**	**89%**
		South Philadelphia	--	--	--
		West Philadelphia	12	2.09 mi (0.46)	11%
DC	*n*=34	Ward 7	81	8.03 mi (0.89)	21%
		North Ward 8	35	5.53 mi (0.85)	9%
		South Ward 8	8	7.50 mi (0.00)	3%
	^b^	Ward 1	67	2.65 mi (0.23)	26%
	^b^	Ward 4	4	3.75 mi (0.10)	0%
	^b^	**Ward 5**	**153**	**4.12 mi (0.20)**	**38%**
Houston	*n*=69	Central Northwest	20	14.72 mi (0.35)	1%
		First Ward	--	--	--
		Greater OST	--	--	--
		Greater Third Ward	37	7.14 mi (0.19)	17%
		Greenway Plaza	--	--	--
		Golfcrest/Belfort	2	8.00 mi (0.00)	0%
		Hobby Airport	4	11.10 mi (1.20)	0%
		Kashmere Gardens	8	13.40 mi (0.00)	1%
		South Park	6	4.80 mi (0.00)	0%
		Sunnyside	21	3.36 mi (0.74)	6%
	^b^	Acres Home	22	18.65 mi (1.28)	9%
	^b^	Greater Hobby Area	3	15.37 mi (0.46)	0%
	^b^	Greater Magnolia Park	72	11.47 mi (1.44)	22%
	^b^	Montrose/Midtown	34	7.61 mi (0.95)	3%
	^b^	Northside/Northline	24	15.25 mi (2.64)	4%
	^b^	Northside Village	49	11.79 mi (1.48)	16%
	^b^	South Houston	34	12.74 mi (0.08)	14%
	^b^	Southwest Houston	7	12.51 mi (3.73)	1%
	^b^	West/Mid-West Houston	25	13.88 mi (1.34)	6%
LA	*n*=85	**Downtown LA**	**82**	**13.70 mi (1.42)**	**31%**
		Long Beach	78	28.64 mi (1.03)	19%
		The Valley	44	14.57 mi (0.57)	16%
		West LA	8	8.00 mi (0.00)	0%
	^b^	Eagle Rock	19	25.00 mi (0.00)	8%
	^b^	Echo Park	4	16.30 mi (0.00)	0%
	^b^	El Sereno	33	22.43 mi (0.17)	14%
	^b^	Highland Park	28	19.21 mi (0.29)	12%

^a^Data are provided as of the first two years of implementation (05-2021 to 04-2023); enrollment is ongoing. High enrollment neighborhoods (i.e., enrolled ≥30% of the sites’ total PWID sample) are in **bold** font

^b^New neighborhoods identified after the pre-implementation landscape analysis

In contrast, new neighborhoods not identified in the pre-implementation landscape analysis were largely initiated in DC, Houston, and LA, where the locations of PWID were more dispersed throughout the cities (Table 2). This required sites to leverage additional data points to identify new neighborhoods and determine the viability of these neighborhoods, as described below. This approach supported the emergence of Downtown LA as a high enrollment neighborhood due to the concentration of PWID found through Project Room Key, a COVID-related housing response for homeless individuals in LA. In contrast, Ward 5 emerged as DC’s high enrollment neighborhood because it provided a safe and easily accessible location through which PWID identified via outreach in other neighborhoods could access the mobile unit using public transportation. Houston covered the largest geographic range, with 16 interconnected implementation neighborhoods, and was the only city without a high enrollment neighborhood.

### Contextual barriers affecting the viability of implementation neighborhoods

Cross-site heterogeneity in sociopolitical and drug use environments affected the viability of some implementation neighborhoods. For example, the geographic diversity of cities required greater driving distances to cover larger geographic regions in Houston and across LA County to reach PWID. As noted, “*LA geography – everything is out of the way – nothing is close – makes it challenging to get places because traffic is between distance”* (Steering Committee Meeting, 03/24/2023). Similarly, proximity to and need for social services in low-income areas resulted in high engagement but low enrollment due to community members seeking access to housing, employment, and healthcare resources. For example, in Houston:

*“Community member stated that most of the drug use in the area is non-injection crack and recommended mobile unit moves further East; stated main problem in the Sunnyside area is poverty, so social services (housing, jobs, ID, crime prevention) are needed more than help for opiate use.”* (Sunnyside, Ecological Observation, 10/18/2021)

Policing and surveillance of PWID were observed across all study sites, particularly the policing of unhoused individuals ramped up in response to election cycles. Exposure to violence was reported across implementation sites, with more frequent reports occurring in Philadelphia, DC, and Houston. An illustrative quote from Philadelphia details, “*more violence and gunshots around the past 6 months. Not affecting participants enrolled yet or ability to recruit, but violence is coming up more*” (IS Team Meeting, 10/07/2022).

Increased police activity, driven by sociopolitical pressures and amplified surveillance efforts, sometimes hindered new enrollments across all sites. Specifically, in NYC and DC, the viability of certain implementation neighborhoods was affected. The East Harlem neighborhood, which had been NYC's exclusive implementation site for over a year, became untenable after an outdoor police command unit was established at a key drug-purchasing route. This sustained law enforcement presence altered drug activity in the area, prompting the NYC site to pursue an alternative implementation neighborhood. In DC, over-policing and 24-hour surveillance efforts linked to ongoing gentrification in Ward 1, 4, 7, and 8 neighborhoods effectively drove PWID from well-established drug-using neighborhoods, signaling a transition away from more open-air drug-use environments. As described*, “Construction and gentrification in the area has pushed out much of the population that injects. We received information that most PWID can be found on the side streets… but most the people who hang out on the main road and wall in front of McDonalds snort or smoke”* (Ward 7, Ecological Observation, 03/18/2022).

Heterogeneity in local drug use environments limited the utility of overdose data to identify viable neighborhoods in areas where PWID are more dispersed. In discussing surveillance data limitations in Philadelphia, one staff described:

*“Starting to go more West Philly. Most folks are not injectors or not injecting opioids. West Philly* (PWID) *are going into Kensington anyway from home to go buy drugs and use* (sterile syringe program) *services. So, Kensington still most high enrollment.”* (Steering Committee Meeting, 01/27/2023)

In Houston’s Sunnyside neighborhood, fatal overdoses did not reflect injection as the primary route of opioid administration. Insight into this was provided by staff who noted, “*A lot of the spaces we are going to that have largely Black communities they are not injecting or they are not using opioids, mostly cocaine”* (Steering Committee Meeting, 08/26/2022). Identifying viable neighborhoods when PWID are more geographically dispersed required additional strategies (e.g., support from Pastors [Houston]) to build rapport among people who use drugs in new neighborhoods and obtain insights on the local drug use environment.

Ecological observations documenting exposure to violence, both persistence and increases, were noted across sites and described as a key barrier to onboarding some implementation sites. For example, in an IS Meeting probing experiences related to safety and well-being, DC staff describe that there are:

*“Increases in violence since the start of study… the team did witness a shooting across the street. This does determine where we will and will not go…Used to be more predictable; violence happened in the evening but it’s happening more in daytime. And yesterday shooting, at 12 noon, 4 people shot. But not too far from* (implementation neighborhood with) *high level of OD* (overdose)*.”* (10/07/2022)

All sites reported encountering opioid users who either refrained from injecting or switched to smoking due to the higher overdose risk with fentanyl in the drug supply. To address challenges in finding PWID in gentrifying neighborhoods, DC used pre-screen surveys delivered by outreach teams to engage with community members and assess the current drug use landscape. As reported:

*“People are just not injecting opioids – opioid use is definitely changing. More cocaine and crack smokers. These* (outreach sites) *are areas driven by opioid deaths. Except that the stimulants are laced with fentanyl. 55% of participants are reporting poly substance use in the past month. Moving from injection to sniffing. The proportion of injectors is lower than before.”* (Steering Committee Meeting, 08/26/2022)

### Contextual facilitators affecting the viability of implementation neighborhoods

Outside of known open drug markets, our IS data revealed that identifying viable implementation neighborhoods in areas with geographically dispersed PWID was supported through a multi-faceted approach. Specifically, local overdose data needed to be augmented by community insights on where opioids and injection behaviors could be found in the neighborhood alongside proximity to built physical environmental factors and local integrated response systems. Through data triangulation, suitable neighborhoods for implementation were pinpointed.

The viability of implementation neighborhoods was improved by identifying factors in the built physical environment, such as local venues in LA and Houston (e.g., gas stations, encampments/apartment complexes) and services systems, particularly public transportation hubs, in Houston and DC. For example, “*Heavy overdoses in the… tent city in Northside Village where we got lots of* (internal) *referrals”* (Steering Committee Meeting, 08/26/2022). These points of connection facilitated outreach and recruitment via greater foot traffic in the neighborhood when venues were known to be associated with drug sales/use, as reflected in Houston’s observations of the Greater Third Ward neighborhood:

*“New Neighborhood. Very tight-knit community, historically low-socioeconomic status and* (racial/ethnic) *minority residents. Location chosen due to very high level of drug activity/sales in the area. Very high foot traffic... 26 visitors which is about 3 times the usual amount of visitors… A few blocks from Metro Light Rail line. New site is at a gas station that is frequented by community members... Community members that stopped by were very welcoming and accepting … stated that we are parked in the right spot for what we are doing.”* (Ecological Observation, 10/18/2022)

Less viable neighborhoods in Houston had convenient freeway access to the implementation sites, yet, these locations were mainly accessible to PWID with personal vehicles, driving from different parts of the city to reach the mobile unit. For example, *"*(Sunnyside is) *not offering candidates that live in the neighborhood. Candidates drive from other neighborhoods (East of Downtown)* (Ecological Observation, 10/07/2021).”

Uniquely, DC leveraged a well-known and busy intersection at a strip mall (the metro center in Ward 5) to establish its strongest implementation site. This strip mall is a trusted area PWID felt safe traveling to meet up with the mobile unit when recruited via outreach teams in neighborhoods where it was deemed unsafe to park the unit for prolonged periods of time. As such, DC staff noted, “*Having a second mobile unit that is smaller is very helpful…* (for) *field safety and security…Smaller unit* (not used to implement integrated care)* is making it easier to get in and out of these places quickly to scope them out*.” (Steering Committee Meeting, 02/10/2023)

### Leveraging local response systems as a real-time data-driven strategy

All sites continued to monitor local overdose data to identify potential implementation sites. Identifying and linking into local overdose and housing response systems was beneficial where there was greater geographic dispersion of PWID. This process helped to identify viable implementation neighborhoods in Houston and LA. Monitoring overdose data from the Houston Fire Department's overdose response unit and the city's homeless outreach team helped identify viable neighborhoods and where to move the mobile unit within existing neighborhoods in real-time.

*“Houston Fire Dept has an outreach worker that follows up with individuals that received care for overdose.* (Our Peer Navigation) *Supervisor rides along with outreach worker and shares neighborhood information with HPTN 094 Team when there are clusters of overdose*s.” (Northside Village Neighborhood, Ecological Observation, 05/02/2022)

*“Planning to move to another parking location within the same neighborhood because the area was listed on the Houston Fire Dept "overdose surveillance list". Participants from the community gathered at mobile unit for moral and emotional support after getting news of recent overdose death of a participant.”* (Magnolia Park Neighborhood, Ecological Observation, 02/21/2022)

While DC's overdose data confirmed overdose hotspots in their implementation neighborhoods, responses to overdose spikes often led to increased police presence and community surveillance, pushing PWID further underground and out of reach of the study team. Following a cluster of ten overdoses, the DC site reported, “*Doing a lot of community work with the Department of Behavioral Health and community-based organizations. We are now part of the rapid response* (to) *overdose spikes…* (It’s become) *public news, by* (the) *7-11, police put remote devices in the areas where there are overdoses”* (Steering Committee Meeting, 02/25/2022).

In comparison, the LA site connected with housing response systems that emerged during the COVID-19 pandemic (e.g., Project Room Key in Downtown Hotels, Tiny Home Villages across LA County). This approach proved more effective in identifying viable implementation neighborhoods than solely relying on local surveillance data.

*“Overdose data and HIV maps took us to Long Beach, but* (the data are) *really not granular. So hard to locate where to actually park.* (When we first moved) *into downtown LA – we followed overdose data to MacArthur Park. It was not useful. There were overdose deaths yes, but they were not our people. Our* (next) *struggle was following these transitional housing units in place - in response to COVID, but* (the housing units) *are staying in response to housing crisis. Find these units - park there, 10% of the population at a given site meet inclusion criteria. So tiny homes at Highland Park 100 units, enrolled 10, Eagle Rock 40 units, enrolled 4. Multiple enrollments in Project Room Key* (Downtown LA) *- originally had 4 sites, the other 2 sites were outside our geographic desired areas.”* (IS Meeting, 08/05/2022)

## Discussion

Our findings illustrate the utility of applying a data-driven approach across heterogeneous geographic, sociopolitical, and drug use landscapes to guide the implementation of INTEGRA, a complex intervention. Local context influenced the effectiveness of surveillance data in identifying suitable neighborhoods for integrated MOUD and HIV care delivery to PWID through mobile units. We identified that surveillance data worked best in neighborhoods with geographically concentrated open-air drug markets rather than areas with dispersed drug access or services. In some cities with geographically dispersed PWID, leveraging local overdose and housing response systems as an additional data source proved beneficial, but not when accompanied by increased police surveillance. In areas where PWID are more geographically dispersed, physical environmental factors like access to public transportation, housing, encampments, and squats were important in determining suitable implementation sites.

We found that shifts in drug markets and sociopolitical factors, including policing and gentrification, can impact the viability of neighborhoods for mobile unit integrated care. Paralleling previous research, the mobile nature of INTEGRA allowed for easier adaptations to these environmental changes compared to traditional fixed-site services [46]. For example, DC utilized public transit infrastructure to direct PWID to a trusted implementation site through targeted screening and outreach to areas affected by gentrification, surveillance, and increased violence. In NYC, Philadelphia, and Houston, leveraging local knowledge of the drug use landscape helped to optimize the reach of INTEGRA within a given neighborhood, while LA developed a synergistic relationship with the County’s emerging transitional housing system. From an implementation and sustainment perspective, these adaptive strategies highlight the importance of building linkages between mobile integrated care and the local service environment; extending the utility of such systems to optimize the reach of HIV and MOUD services in local communities [47]. For example, in areas with high-need or insufficient health service infrastructure, policies that incentivize mobile unit collaborations between integrated care providers and community-based organizations or settings with publicly funded infrastructure (i.e., housing first models, transportation hubs, first responders) could leverage the flexibility of this mobile care model and serve as a blueprint to enhance service accessibility and reach [48–52].

Finally, not all priority neighborhoods identified in the pre-implementation stage were amenable to the mobile delivery of integrated care. Real-time implementation data evidenced numerous challenges, including limited access to parking and escalating risk of violence. Building incentives for local businesses to host medical units has facilitated community access in similar studies [53]. In areas like LA and Houston, navigating large geographic distances between implementation neighborhoods further introduced challenges for sites to ensure continuous access to care during the 26-week intervention window, particularly among PWID with limited transportation access. Such considerations suggest urbanicity may affect the scalability of this approach across different regions of the US [54]. This may speak to the need for multiple mobile units with expanded integrated services that benefit PWID and local community members with similar healthcare needs (e.g., general sexual health screening and PrEP/reproductive care access) to sustain service coverage across geographically dispersed high-need areas [55]. Our findings echo the call from Wagner and colleagues [56], emphasizing the importance of attending to factors in the outer setting in efforts to advance health equity through IS.

This analysis affords important lessons learned for advancing IS research on the mobile delivery of complex interventions [55, 57–59]. Aligned with applications of PRISM [60, 61], this work extends the focus of external environments beyond community resources to include local community dynamics [42] and underscores the utility of a data-driven process to tailor other public health interventions requiring a nuanced understanding of how the outer setting affects implementation outcomes, such as the appropriateness or viability of specific neighborhoods as implementation sites over time and across diverse settings. Specifically, we observed these contextual factors can affect the utility of surveillance data and require iterative assessments during the full implementation period to identify and trial adaptive strategies to optimize the local access and delivery of integrated care. This builds on previous work that deployed COVID services to hotspots based on community-level social vulnerability metrics [62]. Assessments of external community environments can further shed light on issues of health equity across implementation sites, such as local enforcement of policies, policing, and resource allocation that can influence if and how mobile integrated care can be delivered to PWID [63] and similar legally marginalized statuses (e.g., people who engage in sex work or who are undocumented) and minoritized communities. Future analyses will prospectively examine how community environmental factors that affected the viability of implementation neighborhoods (i.e., geographic, drug use, and sociopolitical contexts) might, in turn, affect clinical outcomes of interest, including the adoption and maintenance of ART/PrEP and MOUD treatment at week 26 post-baseline.

The current study is not without limitations. First, as a pragmatic, hybrid, type 1 study [64], we did not systematically assess the viability of all priority neighborhoods identified by HIV and overdose death surveillance data. Nor did we standardize the number of implementation neighborhoods or length of time sites implemented in intervention neighborhoods, limiting the ability to generalize observations across all study sites. Second, these findings do not establish a causal relationship between neighborhood characteristics and neighborhood viability. The presence of the mobile unit/access to integrated care may have indirectly influenced subtle changes in sociopolitical forces that affected the viability of a particular neighborhood. Third, observations were derived from staff and cannot eliminate the potential for observer bias and confounding variables (e.g., the availability of Spanish-speaking staff in Spanish-speaking neighborhoods). Efforts to triangulate observations across multiple sources, both within and across study sites, strengthen the internal validity of our findings. Finally, the heterogeneity within and across study sites strengthens the external validity and potential utility of applying this data-driven approach to delivering INTEGRA across diverse geographic, drug use, and sociopolitical contexts in the US.

## Conclusions

The intersecting opioid and HIV epidemics have accelerated to a crossroads, as syndemic factors increase the complex healthcare needs among PWID in the US and diminish the capacity to engage within a healthcare system that typically siloes access to HIV and addiction care services. Our findings suggest data-driven approaches may provide a pragmatic strategy to guide the real-time implementation of integrated care models in local communities with heterogeneous geographic and drug use landscapes to better meet the treatment needs of PWID.

## Data Availability

The data that support the findings of this study are available from the corresponding author upon reasonable request.

## Abbreviations

ART: Antiretroviral therapy
COVID-19: Coronavirus disease of 2019
DC: District of Columbia
HIV: Human immunodeficiency virus
HPTN: HIV Prevention Trials Network
IS: Implementation science
LA: Los Angeles
MOUD: Medication for opioid use disorder
NYC: New York City
OD: Overdose
PrEP: Preexposure prophylaxis
PRISM: Practical robust implementation sustainability model
PWID: People who inject drugs
RCT: Randomized control trial
US: United States
